# No implant, no solution, lost cases to surgery: orthopedic trauma triage for surgery in an NGO hospital in Sierra Leone

**DOI:** 10.1007/s00402-020-03747-2

**Published:** 2021-01-18

**Authors:** F. Wichlas, V. Hofmann, M. Moursy, G. Strada, C. Deininger

**Affiliations:** 1 Department of Orthopedics and Traumatology, Paracelsus Medical University, Müllner Hauptstrasse 48, 5020 Salzburg, Austria; 2 No Limit Surgery, Ernest-Thun-Strasse 6, 5020 Salzburg, Austria; 3 Emergency NGO, Milan, Italy

**Keywords:** Low-income country, Trauma surgery, Lack of implants, Complex orthopedic injuries, Lost cases, Surgical skills

## Abstract

**Introduction:**

In low-income countries (LIC), international surgeons face the fact that there are patients they cannot treat. The goal of this study was to identify and analyze patients lost to treatment.

**Material and methods:**

We analyzed retrospectively the data of 282 trauma victims from a non-governmental organizational (NGO) hospital in Sierra Leone, Africa. During a 3-month period (10.10.2015–08.01.2016), these patients had 367 injuries and underwent 263 orthopedic surgeries. Despite a clear indication, some patients did not receive surgical treatment. We identified these injuries and the reason why they could not be operated. The anatomic region of the injury was evaluated and if they had a bone or soft tissue defect or were infected.

**Results:**

We identified 95 (25.89%) injuries in 70 patients (47 males; 23 females) that were not be operated. The reasons were lack of specific implants (no implant group; *N* = 33), no treatment strategy for the injury (no solution group; *N* = 29), and patients that were lost (lost patient group; *N* = 33), almost equally distributed by 1/3. In the no implant group were mainly closed fractures and fractures of the pelvis and the proximal femur. The implants needed were locking plates (*N* = 19), proximal femoral nails (*N* = 8), and implants for pelvic surgery (*N* = 6). In the no solution group were nearly all bone (*P* < 0.0000), soft tissue defects (*P* < 0.00001) and infections (*P* = 0.00003) compared to the rest and more open fractures (*P* < 0.00001). In the lost patients group, most fractures were closed (24 out of 33, *P* = 0.033). These fractures were mostly not urgent and were postponed repeatedly.

**Conclusion:**

One quarter of the patients did not receive the surgical treatment needed. Besides acquisition of implants, surgical skills and expertise could be a solution for this issue. Nevertheless, these skills must be passed to local surgeons.

## Introduction

Surgeons in low-income countries (LICs) face high numbers of severe injuries and limited resources [1]. In a place, where even standard care of common fractures is difficult [2], the treatment of serious injuries becomes nearly impossible [3]. The high amount of injuries surpasses the hospitals’ capacity and forces the surgeons and the medical coordinators to triage patients that need treatment the most [4]. While urgent injuries, such as open fractures or compartment syndromes need immediate treatment, closed fractures might be postponed and planned for later surgery. Conservative fracture treatment is a valuable alternative in fracture care. Obviously, the indication for it becomes very large in these circumstances.

Besides the treatment of simple fractures, another therapeutic task requires extensive resources: the reconstruction of complex injuries, such as bone and soft tissue defects [5]. Usually not urgent, because already temporarily stabilized, they require a high surgical expertise and, foremost, time. Not only do these surgeries need to be planned and prepared, but the surgery itself is long. That is when the treatment of these complex injuries possibly interferes with urgent surgeries and the two compete for capacities.

Depending on non-governmental Organization (NGO) hospitals and the LIC, the available implants for osteosynthesis vary widely. While some hospitals lack power drills and image intensifiers in the operation theater (OT), others provide implants necessary for most osteosyntheses. The capacity of operating fractures depends significantly on the availability of instruments and orthopedic implants [6].

The extreme number of injured patients, the amount of complex injuries, and the limited implants, all these factors lead to repeated triage and postponing of patients that might be lost to surgery along the way. The question rises, what kind of injuries might be lost and why they are lost to treatment. In LIC, strategies need to be developed to resolve this problem.

The goal of this study was to identify these lost cases and determine their common characteristics to elaborate rescue strategies regarding local treatment capabilities.

## Materials and methods

### Setting

The patients were analyzed in Freetown, Sierra Leona, Africa. The NGO hospital had 85 beds, 8 intensive care beds without ventilator, 3 OT, an outpatient department (OPD), a room for casting/splinting, and one for physiotherapy.

The hospital’s admission criteria were trauma victims, patients requiring general surgery and pediatric patients. Spinal injuries and patients with posttraumatic deformities were not admitted. These included missed injuries that healed in malalignment.

### Implants

Orthopedic implants available were intramedullary nails, external fixators, K-wires and non-locking plates. The Surgical Implant Generation Network (SIGN Fracture Care International, Richland, WA, USA) intramedullary nail was used interchangeably for femur, tibia, and humerus. The external fixator systems were small and large (Hoffmann II external fixator system and Hoffmann II compact, Stryker Trauma AG, Selzbach, Switzerland, and AO external steel fixator, Depuy Synthes, Oberdorf, Switzerland), the K-wires, cerclage wires, and Ender nails were standard sized steel (1.2–4 mm), and the plates were small and large fragment low contact steel plates (Braun Aesculap, Tuttlingen, Germany).

### Surgeries

Orthopedic operations were run by a five-day routine and for emergencies at night and on weekends. A C-arm was available. The first OT was run by the first author, the second by the general surgeon, and the third by junior surgeons for second and third look soft tissue surgeries. Apart from some SIGN nails, mainly all orthopedic surgeries were performed by the first author due to the lack of local orthopedic surgical expertise.

### Epidemiology

The prospective data acquisition was done for 3 months, from the 10th of October 2015 to the 8th of January 2016.

During that time, 282 patients were admitted having 367 injuries. Of these 282 patients, 273 had 349 fractures and 9 had none. There were 211 adults and 71 children; 205 male and 77 female patients. The left side was fractured 184 times, the right in 150. In 63 patients (22.34%) more than one bone was fractured. The injuries were caused by road traffic accidents (RTA, *N* = 215, 76.24%), falls (*N* = 59, 20.57%), falls from height (*N* = 6, 2.13%), and stab wounds (*N* = 3, 1.06%).

We performed 263 orthopedic surgeries on these 282 trauma patients in 64 days in the orthopedic OT. Open fractures were debrided initially in the OT on admission, with or without external fixation. Hundred and eighty-five patients received one or more osteosyntheses.

The complete dataset of this population has been published before [7].

As the capacity of the hospital was overwhelmed with a high patient inflow, the surgeries had to be triaged on a daily basis. This resulted in injuries that could not be operated, although there was a clear indication for surgery according to western textbooks (LIT-AO Manual rüedi murphy and skeletal trauma (browner, Jupiter, levine, trafton, kretek) tscherne reihe). We identified these injuries and the reason why they could not be operated. The anatomic region of the fracture was evaluated, if they had a bone or soft tissue defect or were infected, and if the fractures were open or closed.

Continuous variables were expressed as means ± standard deviation, whereas categorical variables as percentages (%). Differences for categorical variables were assessed with the *χ*^2^ test. Differences were considered statistically significant if the null hypothesis could be rejected with > 95% confidence (*P* < 0.05).

## Results

We identified 95 (25.89% of 367 injuries) injuries in 70 patients (47 males; 23 females) that could not be operated. The mean age was 32.44 years (range from 4 to 90 years), 61 patients were admitted in the time of data acquisition, and 9 were already in the hospital.

Three reasons for an injury not to be operated were identified:

- No implant
- No solution
- Lost patients

“No implant” means that the specific implant necessary for surgery was not available.

“No solution” means that at that time, a solution could not be elaborated. Mostly, this was due to a lack of time needed for planning, for research of literature, and a different view “out of the box” of an international surgeon under pressure.

“Lost patients” stand for patients that were not urgent, or could not be admitted due to lack of beds, or had an old fracture, or patients in which the reduction of fractures were acceptable, all resulting in repeatedly postponing until treatment became unnecessary or the patients were gone. An acceptable reduction in this context meant the soft tissues were not in danger and the fracture would heal in malunion with all its sequelae such as posttraumatic arthritis, malalignment, deformation and loss of function. Hence, these fractures were a clear indication for surgery by high-income country (HIC) textbooks (lit wie oben) but the reduction was acceptable in these circumstances.

These 3 reasons and whether these injuries were open or closed, had a bone or soft tissue defect, or had an infection are shown on Table 1.

**Table 1:** Listing of the three subgroups and breakdown by type of injury

	Injuries	Open	Closed	Bone defect	Soft tissue defect	Infection
No implant	33	7	26	0	3	0
No solution	29	24	5	13	13	7
Patients lost	33	9	24	1	1	0

The 3 reasons as well as the anatomic regions of the injuries, their bone and soft tissue defects, their infections, and if the fractures were open or closed are shown in Table 2.

**Table 2:** Listing of the three subgroups, classification of fractures according to AO classification and assignment to defined body regions

Region (*N*)	*P*	Implant	No Sol	Lost	BD	STD	Inf	Op	Clo
Pelvic (8)	8	6	0	2	0	1	0	2	6
Pelvic ring, AO 61 (4)		3	0	1	0	1	0	2	2
Acetabulum, AO 62 (3)		3	0	0	0	0	0	0	3
Hip dislocation (1)		0	0	1	0	0	0	0	1
Femoral (20)	18	10	4	6	2	1	0	4	16
Proximal, AO 31 (4)		4	0	0	0	0	0	0	4
Shaft, AO 32 (10)		5	2	3	1	0	0	2	8
Distal, AO 33 (4)		1	2	1	1	1	0	2	2
Patella, AO 34 (2)		0	0	2	0	0	0	0	2
Tibial (35)	31	10	12	13	7	7	4	21	14
Proximal, AO 41 (6)		4	1	1	0	0	1	2	4
Shaft, AO 42 (23)		5	11	7	7	6	3	17	6
Distal, AO 43 (3)		1	0	2	0	1	0	1	2
Malleolar, AO 44 (3)		0	0	3	0	0	0	1	2
Foot (6)	6	0	4	2	3	4	0	6	0
Crushed Foot (4)		0	3	1	3	3	0	4	0
Calcaneus (1)		0	0	1	0	0	0	1	0
Heel Degloving (1)		0	1	0	0	1	0	1	0
Humeral (13)	11	3	6	4	1	1	2	2	11
Proximal, AO 11 (3)		1	2	0	0	0	2	0	3
Shaft, AO 12 (6)		2	1	3	0	0	0	0	6
Distal, AO 13 (2)		0	1	1	1	0	0	1	1
Scapula, AO 14 (1)		0	1	0	0	1	0	1	0
SC Dislocation (1)		0	1	0	0	0	0	0	1
Forearm (7)	7	4	1	2	0	2	0	3	4
Proximal, AO 21 (1)		1	0	0	0	1	0	1	0
Shaft, AO 22 (1)		0	0	1	0	0	0	1	0
Distal, AO 23 (5)		3	1	1	0	1	0	1	4
Hand (5)	5	0	2	3	1	1	0	2	3
Crushed Hand (2)		0	2	0	1	1	0	2	0
Hand (3)		0	0	3	0	0	0	0	3
Mandibula (1)	1	0	0	1	0	0	0	1	0
Total		33	29	33					

*P *patients, *Implant *no implant, *No Sol *no solution, *Lost *lost patients, *BD *bone defect, *STD *soft tissue defect, *Inf *infection, *Op *open fracture, *Clo *closed fracture

Among these patients were 2 children with an osteomyelitis of the proximal humerus (AO 11).

No implant and patients lost were equally the main reason for not being operated (*N* = 33) followed by no solution (*N* = 29). Nevertheless, these reasons were almost equally distributed by 1/3.

No implant made 8.99% of all injuries (*N* = 367). Closed fractures were mainly in this group, compared to the rest (*P* = 0.0026). Most pelvic (6 out of 8) and femoral fractures (11 out of 20, mostly proximal and subtrochanteric fractures) were in this group.

The implants that were required were 19 locking plates for periarticular fractures (femur, tibia, humerus, and forearm), 8 proximal femoral nails for per- and subtrochanteric fractures (AO 31 A and 32 A3.1), and 6 implants and instruments for pelvic surgery. The lack of long drill bits, long screws, and large reconstruction plates made pelvic surgery very difficult. The shaft fractures of long bones (5 tibia and 2 humerus) were placed too far distally or proximally to be nailed.

**Fig. 1:**
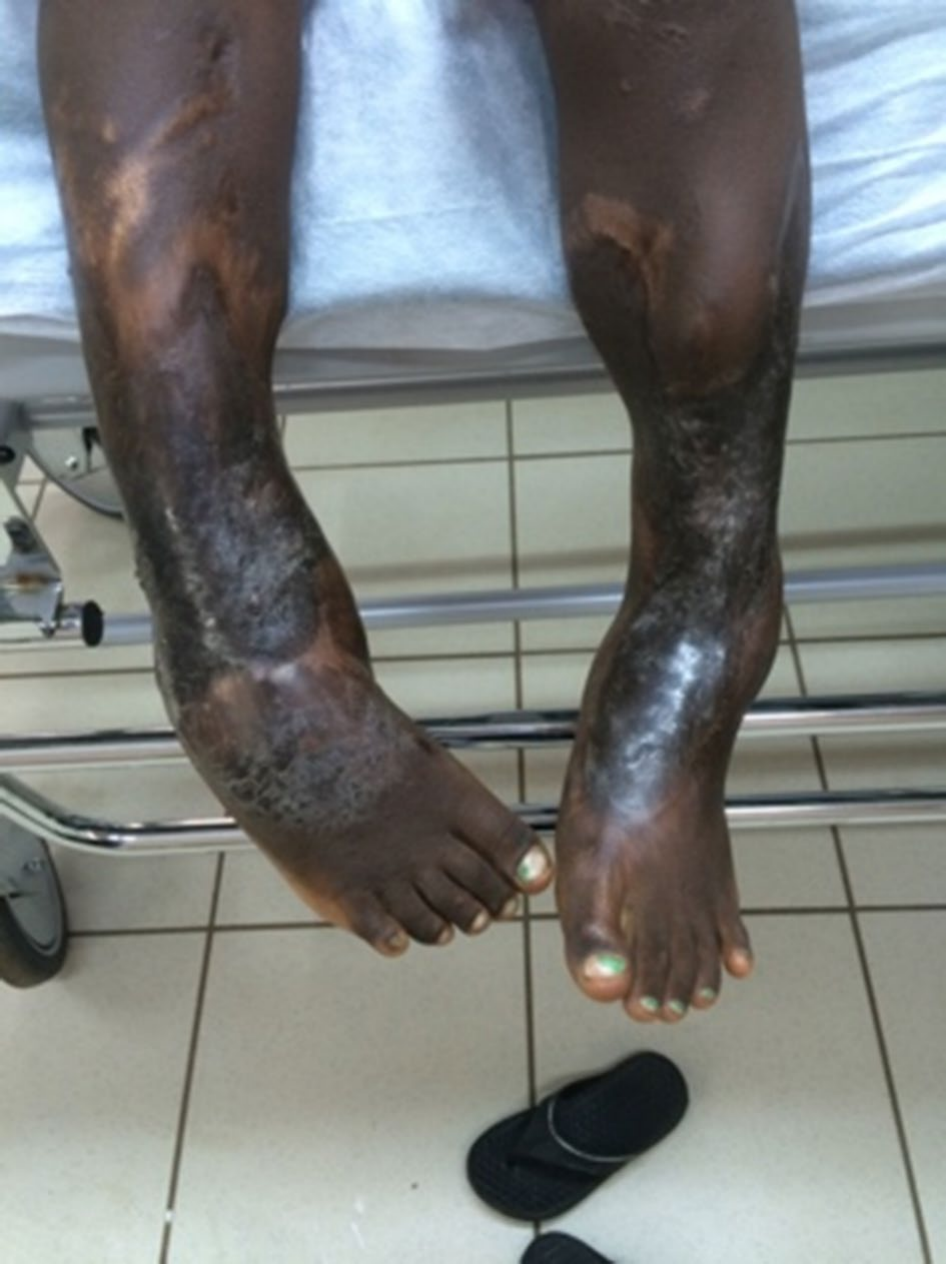
Soft tissue conditions in a 5-year-old girl, one year after rollover trauma with 3-grade open fractures of both lower legs

**Fig. 2:**
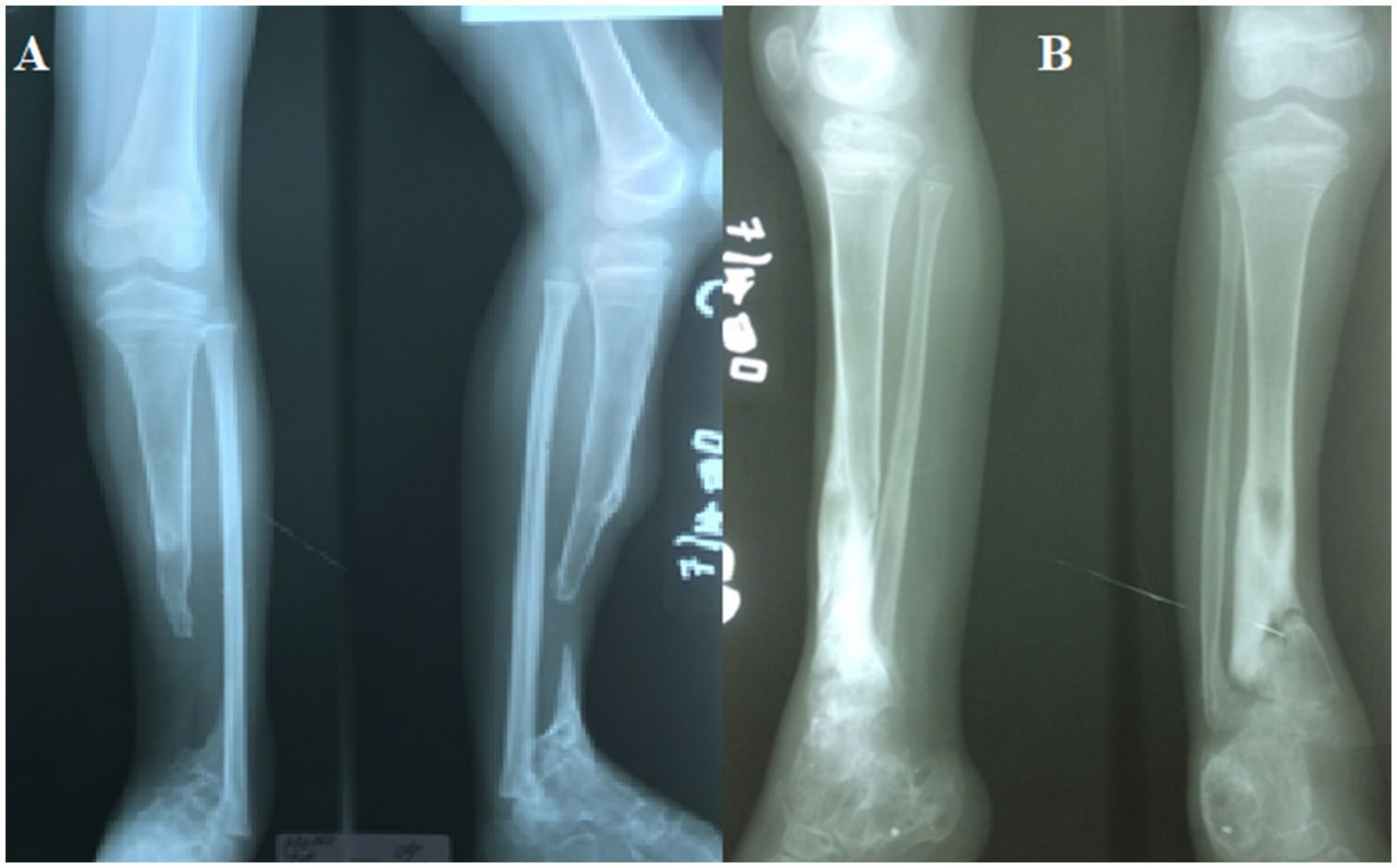
X-rays of both lower legs in 2 planes (A left, B right) 1 year after an open fracture of both lower legs after external fixation and a plaster of Paris

The no solution group made 7.90% of all injuries. Nearly all bone, soft tissue defects and infections were in this group, compared to the rest (*P* < 0.00001 for bone defects, *P* < 0.00001 for soft tissue defects, *P* = 0.00003 for infections). There were more open fractures in the no solution group than in the rest (*P* < 0.00001). In this group were 11 of 23 tibial shaft fractures that had 7 bone defects and 6 soft tissue defects. One such case is shown with an example of a 5-year-old girl with complex fractures of both lower extremities. The lower legs were overrolled by a car. She was initially admitted one year before our mission with an open fracture of both lower legs tretaed by external fixation and a plaster of Paris. After external fixation and plaster of Paris dressing, her soft tissues had finally healed (Fig. 1). As you can see in Fig. 2, she has a tibial non-union on the right side and bony tibial defect on the left. In our setting, we were not able to develop a solution strategy for the diagnoses. Complex reconstructive surgery would probably have a high complication rate and required valuable time-capacities.

The lost group made 8.99% of all injuries, most fractures were closed (24 out of 33, *P* = 0.033), and the fractures had one bone and one soft tissue defect only. These fractures had in common that they were not urgent, had no priority and were postponed repeatedly.

Most of them (*N* = 29) were fractures with clear indications for surgical treatment but not urgently. Other fractures were old fractures that needed a planned surgery (*N* = 4).

## Conclusion

A quarter of all injuries (25.89%) were not operated in this scenario. According to the authors, these injuries should have been operated but due to the mentioned findings, could not.

Three main reasons could be identified in this study population accounting for nearly one third each.

The lack of implants does not seem surprising in LIC [8], although this hospital was comparably well equipped. Without the high number of external fixators (approximately 70), the unlimited use of K-wires and SIGN nails, the amount of not operated patients would have been much higher. As these fractures were mainly closed, the external fixator was not the first choice because it was saved for open fractures. No implant was relevant for 9.26% of all injuries. There are two ways to overcome this problem: acquisition of implants or surgical expertise for improvisation.

In our opinion, the most needed implants for this hospital were locking plates. Nevertheless, orthopedic trauma surgery existed long before the development of these fixation systems, but the fact that a good bone–screw interface is not needed is invaluable for complex articular fractures [9]. Our practical experience in Sierra Leone showed weak bone quality, even in younger patients. The bone strength often resembled to osteoporotic bone, which might be caused by malnutrition [10]. Conventional, non-locking, plates were used, but the screws had a limited purchase in the bone. However, the need for locking plates has to be seen in context because this hospital is exceptionally well equipped compared to other NGO hospitals, where even image intensifiers and power tools are missing.

The fact that the lack of a special implant, the locking plate, has an impact on the treatment reflects the problem of surgeons from HIC. The surgeons depend more on technical tools than on the surgical craft, especially in orthopedic trauma surgery [11]. It was difficult for us to change the view, create solution strategies, even more under the high work load condition. Because these implants are expensive and unaffordable for most NGOs, surgical skills and improvisation only can be an alternative to conservative therapy. That is why, in the course of this mission in Sierra Leone, we learned to use the implants available “off-label”. K-wires were used for tibial shaft fractures in combination with plaster of Paris in adults and external fixators instead of locking plates. Being used for merely every bone, especially the use of external fixators is indispensable for NGO hospitals and their indication seems to be widened even nowadays in HIC [12–15].

In the case of pelvic fractures, the lack of implants was evident, but the problems were identified only while operating on an acetabular fracture. There were no long screws, no long drill bits, no carbon table, and the reconstruction plates were too short. Two operating tables were tied together to permit intraoperative X-ray control. The assistant had never seen such surgery before, as did the nurse, and the surgery lasted 8 h. In that time, 4–6 urgent cases could have been operated. Two cephalomedullary nails were performed before the medullary reamer was broken. From that day on, no per- and subtrochanteric fractures (*N* = 8) could be operated and were put in traction.

For severe fractures, no solution could be elaborated in time. These were mainly open fractures with bone and soft tissue defects, made 7.90% of all injuries, and require complex surgical reconstruction. In HIC, they would be referred to specialized orthopedic trauma centers where orthopedic and plastic surgeons would be involved in the treatment [16, 17]. In LIC, these complex surgeries would have blocked the OT for a day and the postoperative management would have to deal with comparably high failure rates [18]. Although modern surgery provides elegant solutions [19], in this time-consuming setting even a plan could not be made.

These cases highly depend on the treating surgeon and the situation. A surgeon with a different skillset at another time point, maybe with less patient inflow, could have treated more of these patients. Severe bone and soft tissue defects often need reconstructive surgery using osteomusculocutaneous flaps, increasingly used in LIC [18].

Severe soft tissue and bone defects can be dealt with surgical expertise only.

Lost patients (8.72%) were dismissed by more urgent cases. Although the OT capacities were sufficient, the limited number of surgeons in the hospital leads to these compromises and triages. An increase in surgical personnel could resolve this mismatch. As most of these patients have simple fractures and standard osteosynthesis such as nailing or external fixators would suffice for treatment, only a basic orthopedic traumatological expertise is required. This could be provided by local surgeons, when available, or by young specialist from HIC. The lack of surgical expertise is daily business in LIC [20–22]. However, the fact that the injuries were lost to surgery does not mean that they were not treated. Casting and traction were the alternative to surgery.

While the problem of no implant could be solved by increasing the material resources or surgical improvisation, the solution for the other groups could be solved by specialized personnel only. In this hospital, the OT capacities and the available instruments were sufficient, but apart from international surgeons, there was no orthopedic trauma expertise. However, more than bringing the expertise temporarily to LIC by international surgeons, it is necessary to transfer the expertise to the local surgeons. This requires the presence of local surgeons, willing to learn and stay in the hospital, at least for some time. While the surgeons in some LIC are eager to learn and are motivated, others are not. In Sierra Leone, the local doctors were mainly on rotation and had no intention to become surgeons. While diagnostic and preoperative pathways are managed efficiently by the local staff, the treatment ends at the OT door. International surgeons handling the workload and local surgeons ready for private practice will result in a temporary management of hospital capacities only. NGO have surely understood this point, but the implementation was not successful everywhere [23, 24]. A well-equipped hospital with low surgical expertise probably is not as effective as a low equipped hospital with well trained staff. It is better to invest in people than in infrastructure, or implants. The fact that teaching is essential has been widely accepted [25–28] and material resources have to be used wisely [29, 30]. For the hospital in Sierra Leone, our proposed concept would be to deploy two international surgeons for mission. One experienced senior surgeon treating complex cases and serving as backup for an experienced junior surgeon treating acute injuries in collaboration with local surgeons. The local surgeons could learn to operate acute injuries and later complex reconstructions and the international junior surgeon could be won for this kind of medicine. This could help to solve the problem of the NGO surgeons becoming too old for the mission.

More than anywhere else, skills and surgical expertise are the key for the successful trauma treatment in LIC. They could solve the treatment of complex cases, a high turnover of acute patients, and replace missing implants. International surgeons can provide this knowledge, but must transfer it to local surgeons.
